# Overcoming challenges to sustainable immunization financing: early experiences from GAVI graduating countries

**DOI:** 10.1093/heapol/czu003

**Published:** 2014-02-08

**Authors:** Helen Saxenian, Robert Hecht, Miloud Kaddar, Sarah Schmitt, Theresa Ryckman, Santiago Cornejo

**Affiliations:** ^1^Consultant to Results for Development Institute, Washington, DC 20005, USA, ^2^Results for Development Institute, Washington, DC 20005, USA, ^3^World Health Organization, 1211 Geneva, Switzerland, ^4^Consultant to World Health Organization, 1211 Geneva, Switzerland, ^5^GAVI Alliance, 1202 Geneva, Switzerland

**Keywords:** Immunization, middle-income countries, health financing, donor assistance policies, GAVI, vaccines, national self-sufficiency

## Abstract

Over the 5-year period ending in 2018, 16 countries with a combined birth cohort of over 6 million infants requiring life-saving immunizations are scheduled to transition (graduate) from outside financial and technical support for a number of their essential vaccines. This support has been provided over the past decade by the GAVI Alliance. Will these 16 countries be able to continue to sustain these vaccination efforts? To address this issue, GAVI and its partners are supporting transition planning, entailing country assessments of readiness to graduate and intensive dialogue with national officials to ensure a smooth transition process. This approach was piloted in Bhutan, Republic of Congo, Georgia, Moldova and Mongolia in 2012. The pilot showed that graduating countries are highly heterogeneous in their capacity to assume responsibility for their immunization programmes. Although all possess certain strengths, each country displayed weaknesses in some of the following areas: budgeting for vaccine purchase, national procurement practices, performance of national regulatory agencies, and technical capacity for vaccine planning and advocacy. The 2012 pilot experience further demonstrated the value of transition planning processes and tools. As a result, GAVI has decided to continue with transition planning in 2013 and beyond. As the graduation process advances, GAVI and graduating countries should continue to contribute to global collective thinking about how developing countries can successfully end their dependence on donor aid and achieve self-sufficiency.

**KEY MESSAGE**

- Sixteen countries are currently scheduled to ‘graduate’ from GAVI assistance by 2018. Five of these countries were chosen for ‘transition planning’ support from GAVI and other partners in 2012. Through country assessments, a variety of challenges were identified in transitioning away from GAVI assistance towards national self-sufficiency, including concerns relating to financial sustainability, sound procurement practices, effective national regulatory agencies, and adequate capacity for immunization planning and advocacy. Lessons learned from these five country experiences will help improve future assistance to graduating countries, as well as similar transitions in other areas, such as AIDS, malaria and family planning.

## Introduction

Since its founding in 2000, the GAVI Alliance has provided vaccines, supplies and programmatic support designed to save the lives of young children in over 75 of the world’s poorest developing countries. As of June 2013, GAVI had committed US$8.4 billion in assistance to these countries over 2000–16, resulting in 370 million additional children immunized by the end of 2012 and over 5.5 million averted future deaths from vaccine-preventable diseases (GAVI Alliance 2013b).

GAVI has long been concerned with the sustainability of immunization programmes and their benefits, an issue that is coming to the fore as countries cross the income eligibility threshold and start to graduate from GAVI assistance. GAVI’s graduation process is designed to ramp up domestic co-financing of these vaccines while GAVI financing slowly phases out over several years, so that once GAVI support ends, the country will be able to fully fund these vaccines (and associated costs) in a fiscally sustainable way.

GAVI’s ongoing experience with country graduation is important, not only because immunization for millions of children is at stake but also because GAVI is one of the few global development programmes that is attempting to systematically move countries towards financial self-sufficiency. Although the Global Fund and the World Bank have policies on income and eligibility, graduation from these institutions does not begin until countries reach higher income levels (Global Fund to Fight AIDS, Tuberculosis and Malaria 2011; Heckelman *et al.* 2011; World Bank 2013), and the World Bank does not set an explicit income threshold beyond which countries are cut off from borrowing.^1^ The US President’s Emergency Plan for AIDS Relief (PEPFAR) is currently attempting to transition some countries away from predominantly US funding towards greater domestic financing. However, no clear trigger point for graduation has yet been established (Institute of Medicine 2013).

Although there are a few reviews of graduation from other development programmes (Bertrand 2011), little has been done to synthesize these experiences across programmes. Documentation of GAVI’s efforts may therefore yield valuable lessons and contribute to the larger emerging literature on graduation.

This article describes GAVI’s recent collaboration with global partners and graduating countries to identify obstacles and shape solutions for achieving a smooth graduation. In 2012, GAVI tested an approach to graduation planning in five countries, and found that proactive engagement is likely to be needed in many, if not all, graduating countries.

The article lays out the process that was followed in 2012, and highlights common and country-specific graduation challenges. Although there may be other dimensions of successful graduation, this article focuses on three essential components of GAVI’s approach: (1) full financing of immunization programmes with sustainable domestic resources, (2) country management of vaccine supply and procurement, and (3) development of sound decision-making processes to strengthen immunization services and prioritize future vaccine introductions.

### Recent policy changes on GAVI eligibility and co-financing are driving the graduation process

In January 2011, GAVI established a country-eligibility threshold of US$1500 gross national income (GNI) per capita, with annual adjustments thereafter in order to remain constant in real terms. Each year the most recent World Bank GNI per capita estimates are used to determine which countries have crossed the eligibility threshold. These countries become ineligible to make applications for additional new vaccines. However, GAVI support continues for a time-limited period for the vaccines that GAVI is already financing or has approved in that country.

The first year of ineligibility is considered a grace year and no change is made in the ‘co-financing requirement’—the contribution countries make towards GAVI-supported vaccines. The co-financing requirement starts at US$0.20 per dose for the poorest countries—just a small fraction of the actual cost of the vaccine (GAVI Alliance 2013a). As a country’s income per capita reaches a higher intermediate category, its co-financing requirement increases by 15% per annum. Once a country enters the graduation process, its co-financing requirement is ramped up rapidly so that by the fifth year, countries fully finance their vaccines (Saxenian *et al.* 2011).

### The diverse group of graduating countries and overall financial challenge

The change in GAVI’s eligibility policy in 2011 resulted in a surge in the numbers of countries graduating. As of January 2012, 16 countries started the graduation process. Following this initial surge, the pace of graduation is expected to slow, with the number of countries crossing the eligibility threshold annually varying between 1 and 3, based on projections of national income growth. GAVI’s largest country—India—is likely to cross the eligibility threshold in the next few years.

Within this 2012 cohort of 16 GAVI graduating countries, Cuba and Ukraine have no ongoing or pending vaccine support from GAVI. The remaining 14 countries are diverse in size of birth cohorts, GNI per capita and public government spending on health per capita. Kiribati’s annual birth cohort is <5000, whereas Indonesia’s is 4.3 million (see Table 1). Honduras’ 2011 GNI per capita was US$1980 whereas Azerbaijan’s reached US$5290. Per capita health spending ranges from US$32 in Indonesia to US$158 in Guyana.

**Table 1:** Key characteristics, graduating countries, ordered by population

	Mid-2012 (1000s)		2011			2014–18
	Population	Birth cohort	GNI per capita ($)	Government expenditures on health per capita ($)	GDP growth (%)	Projected annual average GDP growth (%)
Indonesia	244 765	4270	2940	32	6.5	6.5
Sri Lanka	21 559	362	2580	43	8.2	6.6
Angola	20 220	817	3830	115	3.9	6.0
Bolivia	10 321	266	2020	84	5.2	5.0
Azerbaijan	9543	184	5290	77	0.1	4.3
Honduras	7985	206	1980	89^a^	3.7	3.0
Georgia	4450	50	2860	57	7.2	6.0
Congo	4256	148	2250	59	3.4	8.5
Moldova	3602	42	1980	102	6.8	4.8
Armenia	3198	47	3360	51	4.7	4.3
Mongolia	2864	65	2310	92	17.5	9.0
Guyana	763	13	2900^b^	158	5.4	4.4
Bhutan	756	15	2130	78	8.5	10.5
Kiribati	101	<5	2030	142	2.0	2.0

*Sources:* UN Population Prospects. Updated 28 June 2011; cited 18 December 2012. Online at: http://esa.un.org/wpp/Excel-Data/population.htm.

World Bank World Development Indicators. Updated 16 April 2013; cited 4 June 2013. Online at: http://data.worldbank.org/data-catalog/world-development-indicators.

WHO National Health Accounts Health Expenditures Database. Updated 2013; cited 4 June 2013. Online at: http://apps.who.int/nha/database/DataExplorerRegime.aspx.

IMF (2013).

^a^Data are for 2009, not 2011.

^b^Data are for 2010, not 2011.

Economic growth in each country will impact the fiscal space—the domestic public sector resources—available to finance these vaccines. Table 1 shows real gross domestic product (GDP) growth rates to 2018 for the graduating countries, according to the International Monetary Fund (IMF) forecasts. Angola, Bhutan, Bolivia, Georgia, Indonesia, Mongolia and Sri Lanka are projected to grow rapidly. Growth estimates are more modest but still good for Armenia and Azerbaijan, and are somewhat lower for Honduras and Kiribati.

**Figure 1:**
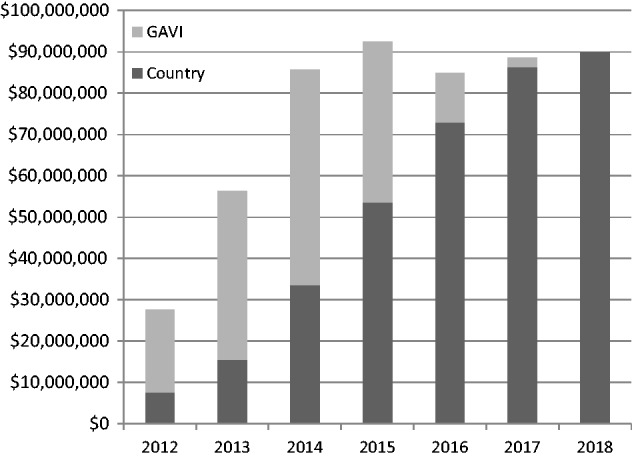
Country and GAVI financing for GAVI-supported vaccines for the 14 graduating countries, 2012–18 (US$). *Source:* GAVI Alliance, estimates as of 26 September 2013. *Note:* These estimates are based on introduction dates and doses from GAVI’s adjusted demand forecast. For vaccines introduced in 2012, GAVI’s last year of support would be 2015. For introduction in 2013, GAVI’s last year of support is 2016. For introduction in 2014, GAVI’s last year of support is 2017.

Overall, the 14 countries are expected to face varying challenges in meeting the costs of their new GAVI-supported vaccines, as external support from GAVI is phased out (see Figure 1). Total required funding for new vaccines (and injection supplies) is anticipated to grow from under US$30 million in 2012 to almost US$90 million by 2018. Funding from GAVI will peak in 2014, at US$52 million, and will then decline to zero by 2018. The financial resources required from the 14 graduating country governments will therefore need to increase from about US$8 million in 2012 to US$90 million in 2018. These projections assume that countries will obtain GAVI prices after GAVI support ends, although alternative country-specific price assumptions were used in discussions with countries. In addition, countries will need to continue to finance non-vaccine immunization costs, such as health workers, transport, demand creation and community mobilization activities, and the supply chain for vaccines.

Financial projections for all 14 countries are shown in Table 2. More detailed analysis was also carried out for selected countries such as Mongolia and Georgia, taking into account different scenarios for timing of vaccine introductions and vaccine prices (Results for Development 2013a,b).

**Table 2:** Projected GAVI contribution and country co-financing for GAVI new vaccine support: 2012–18 (US$)

Country	2012	2013	2014	2015	2016	2017	2018
Angola							
Country	2 267 799	6 933 500	9 536 500	17 327 500	25 494 500	30 988 000	34 542 500
GAVI	4 669 500	11 969 500	13 225 500	13 387 000	2 565 500	1 567 000	0
Armenia							
Country	193 804	292 500	494 000	620 000	848 000	953 500	1 082 000
GAVI	563 500	444 500	1 037 500	742 500	262 500	166 500	0
Azerbaijan							
Country	1 224 450	940 000	1 673 000	1 910 000	2 606 500	3 105 000	3 028 500
GAVI	387 000	3 607 500	2 389 000	1 827 500	413 500	0	0
Bhutan							
Country	39 068	51 500	91 500	93 000	130 500	132 500	133 500
GAVI	98 000	105 000	74 000	38 500	0	0	0
Bolivia							
Country	730 675	614 000	2 058 500	3 073 500	3 804 500	5 083 000	5 134 000
GAVI	1 192 500	1 683 500	3 400 000	2 610 000	881 000	0	0
Congo							
Country	563 712	1 506 000	2 382 000	2 986 000	4 034 500	4 349 500	4 513 500
GAVI	3 490 000	3 979 000	3 061 000	2 273 000	400 000	230 000	0
Georgia							
Country	239 941	299 000	571 500	824 000	1 229 000	1 448 500	1 710 000
GAVI	650 500	545 500	904 500	974 500	369 500	250 000	0
Guyana							
Country	36 447	87 500	133 000	240 500	288 500	378 000	365 000
GAVI	603 000	397 000	232 500	214 000	93 500	0	0
Honduras							
Country	1 088 385	1 467 500	2 042 000	2 708 500	3 572 000	3 578 500	3 365 000
GAVI	5 084 000	3 886 500	2 484 000	2 057 000	0	0	0
Indonesia							
Country	—	2 088 500	11 787 500	20 765 500	27 420 500	32 638 000	32 314 500
GAVI	—	10 024 000	23 931 500	13 843 500	6 855 500	0	0
Kiribati							
Country	15 475	24 000	17 000	37 000	51 500	61 500	60 000
GAVI	15 500	89 500	36 500	38 500	9 500	0	0
Moldova							
Country	154 092	283 500	489 500	728 500	1 002 500	1 149 500	1 116 000
GAVI	482 000	762 500	816 000	642 500	136 000	0	0
Mongolia							
Country	129 985	266 500	424 000	489 500	658 000	668 000	676 000
GAVI	428 500	544 000	307 000	171 500	0	0	0
Sri Lanka							
Country	943 752	646 500	1 928 500	1 796 500	1 823 500	1 906 000	1 860 500
GAVI	2 313 000	2 811 000	271 500	68 500	0	0	0
Total							
Country	7 627 585	15 500 500	33 628 500	53 600 000	72 964 000	86 439 500	89 901 000
GAVI	19 977 000	40 849 000	52 170 500	38 888 500	11 986 500	2 213 500	0

*Source*: GAVI Alliance, estimates as of 26 September 2013.

*Note*: These estimates are based on introduction dates and doses from GAVI’s adjusted demand forecast. For vaccines introduced by 2012, GAVI’s last year of support is 2015. For introduction in 2013, GAVI’s last year of support is 2016. For introduction in 2014, GAVI’s last year of support is 2017.

**Figure 2:**
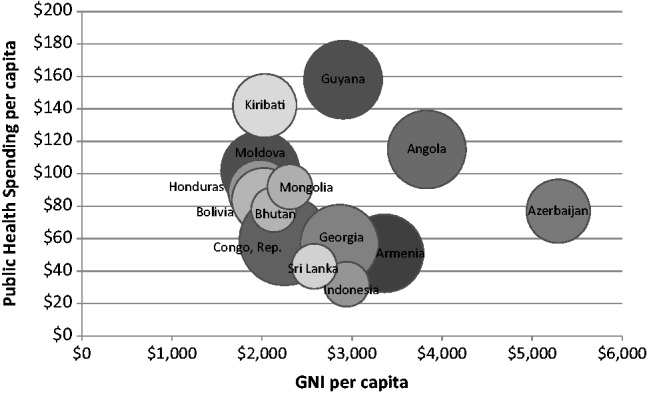
Countries graduating from GAVI support: 2011 GNI per capita, 2011 public spending on health per capita, and number of new vaccines adopted or pending adoption with GAVI support. *Note:* The smallest bubble represents one vaccine adopted or pending adoption with GAVI support; the largest represents four vaccines.

The funds that graduating countries will need to budget for vaccines will depend on the number of new vaccines they have introduced with GAVI support, the quantity of doses required and initially on co-financing requirements. After GAVI financing ends, domestic resource needs will also be driven by the price of the particular vaccine for its specific presentation (single dose vs multi-dose vials, in liquid vs freeze-dried form, etc.). Four of the 14 countries have GAVI funding for only one vaccine—pentavalent (see Figure 2); four have adopted two vaccines with GAVI financing, and five have GAVI support for three vaccines. The Republic of Congo has the largest bubble in Figure 2, as it has obtained GAVI funds to introduce four vaccines.

As a first approximation, affordability can be analysed by considering the number of new vaccines being adopted and the current level of government health expenditure. By this measure, one can foresee challenges in the Republic of Congo, which has relatively low government spending on health per capita (US$59) and is adopting the largest number of new vaccines. In contrast, even though Sri Lanka has lower government spending on health per capita (US$43), it has only one new vaccine adoption to incorporate into its budget.

## Methods

Early in 2012, the GAVI Alliance endorsed a framework to guide country assessments and transitions (GAVI Alliance and World Health Organization, unpublished data), and called on GAVI and its partners to work on ‘transition planning’ with a subgroup of countries. Transition planning was to include: review of key country documents;^2^ visits to the countries to work with government and partner staff to collect additional data; preparation of financial projections and other analyses; identification of issues and options most pertinent to graduation; preparation of a report for government on the main findings and recommendations; and creation of a plan to monitor and follow up on agreed actions.

GAVI selected six countries for transition planning in 2012: Angola, Bhutan, Republic of Congo, Georgia, Moldova and Mongolia. Fiscal space analysis conducted during GAVI’s co-financing policy revision flagged that the Republic of Congo and Moldova might experience the greatest challenges in budgeting for increased immunization spending. Bhutan and Mongolia were selected due to concerns that the countries were not adequately informed about co-financing requirements and phase-out of GAVI support. Georgia was chosen in part because it was felt that ongoing health reforms and related privatization of government health services might adversely affect immunization programmes and financing. Angola was prioritized because of its relative lack of experience with co-financing and the large numbers of vaccines it is adopting.

Transition planning was carried out in five of the six countries in 2012, with work in Angola postponed to 2013. Transition planning teams were made up of experts in financing, procurement and GAVI procedures, and included staff from WHO, the GAVI Secretariat, and specialists from other organizations,^3^ working with country counterparts.

To assess country readiness to graduate successfully and to elaborate transition plans for each country, we used a simple theoretical framework grounded in the literature on sustainability (IMF 2013). Immunization programmes, like many essential health services, need to be maintained over many decades in order to derive important life-saving benefits. This requires a strong immunization system composed of (1) service delivery platforms (government, Non Government Organization (NGO) and private service providers) that are accessible to the entire population, (2) sound policies and institutions (ministries of health, networks of non-government providers, medical associations, etc.) and (3) adequate and predictable funding to cover the costs of vaccines, personnel, cold chain and other inputs. This system in turn requires a range of resources—skilled workers, know how, funding, political commitment and accountability mechanisms—to ensure that systems function efficiently over many years. Our assessment and recommendations for transition focused on these categories of resources and their current and likely future availability.

## Results and discussion

### Fully financing vaccines from government budgets

One of the key transition planning tasks was to develop detailed projections with country governments of the vaccine funding requirements from 2012 to 2018. These estimates were then compared with projected government spending on health to assess the feasibility of covering the additional costs from domestic sources. For example, in Georgia, government vaccine expenditures were projected to nearly double, from US$1.62 million in 2012 to US$3.22 million in 2017 (Results for Development 2013a). However, within the context of projected government health spending, this amounts to 0.5% of government health spending in 2012, rising to 0.7% in 2016/17. Georgia has prioritized immunization, and the transition planning team concluded that the government should be able to finance this increase as long as it maintains its political commitment.

Bhutan and Mongolia are introducing or piloting other vaccines in addition to GAVI-supported vaccines. Bhutan is receiving time-limited external support from a manufacturer and then the Australian Cervical Cancer Foundation for the human papillomavirus vaccine (HPV). Bhutan was the only country which has obtained support for all its routine vaccines from an external donor (Japan Committee ‘Vaccines for the World’s Children’). This funding is on a year-to-year basis, and the transition planning team highlighted the need for Bhutan to be ready to finance and procure these vaccines, should this external funding end.

Mongolia’s HPV vaccine and delivery costs are being temporarily paid for by the US government’s Millennium Challenge Account and the manufacturer. Mongolia is also piloting hepatitis A vaccine introduction financed from the government budget. In multiple adoption and financing scenarios for Mongolia, hepatitis A vaccine absorbed the single largest share of projected vaccine costs (Results for Development 2013b). This finding was surprising to the government and partners, and may spur another examination of vaccine introduction priorities and alternative approaches to addressing hepatitis A.

Moldova has modest economic growth prospects, and as GAVI support is phased out, Moldova’s co-financing is projected to rise from about US$50 000 in 2011 to US$1.1 million in 2018. The government indicated that it was working to create the needed budgetary space. This will be challenging and require careful monitoring, as the percentage increase is significant and Moldova may also be facing a loss of external financing for other key health programmes to control its AIDS and tuberculosis epidemics.

For the Republic of Congo, GAVI is supporting pentavalent, pneumococcal and yellow fever vaccines, and has approved the introduction of rotavirus vaccine. To pay for these four vaccines, Congo’s co-financing will need to increase from about US$90 000 in 2011 to US$3.0 million in 2015 and US$4.0 million in 2016. This is estimated to account for 0.8% of the Ministry of Health’s budget in 2015 and 1.0% in 2016. The government and its partners were optimistic about the government’s ability to fund this increase. The transition planning team nevertheless expressed its concerns about the effectiveness of government to ensure that the required funds are budgeted, approved and channelled to the health ministry. Congo’s default in 2012 on its GAVI co-financing payments highlighted the magnitude of the problem.

In general, countries had not carried out detailed financial projections of vaccine costs by funding source. Government staff indicated that the analyses conducted as part of the transition planning were therefore useful. They stressed concerns over the uncertainty of vaccine prices once GAVI assistance ends.^4^ Although some vaccine manufacturers have indicated that they will continue to provide ‘GAVI prices’ to graduating countries, the prices these governments actually pay will depend on several factors, including global market dynamics, the policies adopted by manufacturers based in part on discussions with GAVI, WHO and United Nations Children's Fund (UNICEF), and the vaccine presentation selected and procurement methods followed by each country.

GAVI has been seeking commitments from manufacturers to provide ‘GAVI prices’ to graduates, and has already negotiated continued low prices for graduated countries for pentavalent, pneumococcal and rotavirus vaccines. The process through which graduating countries will access these prices and the duration of their validity are still being worked out. UNICEF’s vaccine procurement tenders on GAVI’s behalf now include a request for access to GAVI pricing for graduated countries. The first such tender with this condition was for rotavirus vaccine, but only applies to countries which have introduced rotavirus with GAVI support. There is also a special arrangement for pneumococcal vaccines, under which graduating countries that did not apply for GAVI assistance while eligible can still gain access to the Advance Market Commitment maximum ‘tail price’ of US$3.50 per dose (the current price is slightly lower) through the UNICEF Supply Division (SD) (UNICEF Supply Division 2012), using their own financing up to the year 2020 (GAVI Alliance 2013b).

### Procurement management

As part of transition planning, countries need to determine how they will handle vaccine procurement once GAVI support ends. As discussed earlier, GAVI and UNICEF are working with manufacturers to determine whether GAVI prices could be made available to countries post-graduation. These negotiated prices may require that graduated countries continue to use the procurement services of the UNICEF SD. In this case, graduating countries will need to verify whether country regulations permit use of an external procurement agency like UNICEF when national budgets are involved, and potentially modify these regulations.

All five countries studied in 2012 are procuring their GAVI-supported vaccines through the UNICEF SD. Bhutan, the Republic of Congo, Georgia and Mongolia also use the SD for their routine vaccines. Georgia, Moldova and Mongolia carry out some direct procurement of non-GAVI vaccines. All three countries have faced supply and pricing issues with direct procurement. Moldova directly purchases routine vaccines at significantly higher prices—in some cases more than double the prices of those offered through UNICEF—and has experienced greater year-to-year price fluctuations (A Unsatii, unpublished data). In Mongolia, the government was unaware that some of the vaccines that it was procuring directly are offered at lower prices through UNICEF. Switching to UNICEF procurement could thus result in access to higher quality vaccines and to financial savings.

Should Ministries of Health choose not to use the UNICEF SD, it will still be important for them to ensure that vaccine procurement methods result in competitive prices for high quality products. This may be difficult to achieve for the small markets that a number of these countries represent. In addition, direct national vaccine procurement requires specialized market knowledge and skills that still need to be built in some graduating countries.

Countries also need to incorporate product selection in their procurement planning. Although countries have the opportunity to request a particular presentation for a vaccine when applying for GAVI support, this does not occur in most cases, and thus the final decision on what the country receives is largely determined by the UNICEF SD. Most countries visited were not well informed about the multiple presentations (number of doses per vial, cold storage requirements, recommended doses per child, etc.) available for a given vaccine and their financial implications.

The first country visits in the transition planning exercise revealed a possible drawback with the way GAVI was calculating country co-financing requirements. Under the revised co-financing policy adopted in 2011, graduating countries are expected to pay a percentage of UNICEF’s weighted average price (WAP) for the vaccine during the graduation process, with that percentage steadily rising until GAVI support ends. At graduation, however, the countries are responsible for 100% of the actual price of the product and presentation selected. It became clear during the country visits that calculating co-financing requirements based on the current price for the actual product and presentation would better prepare countries for GAVI graduation. As a step towards addressing this issue, GAVI changed the co-financing requirement calculation in 2012 to be a percentage of UNICEF’s WAP by vaccine presentation to account for the fact that single dose presentations can have a significantly higher price than multi-dose presentations.

### Strengthening the national regulatory agency and building capacity for vaccine planning and advocacy

The transition teams examined the status of the countries’ national regulatory agencies (NRA) and the ability of the graduating countries to handle their own vaccine planning, advocacy and other technical tasks.

The risks from poor quality vaccines are considerable: adverse events can destroy public confidence in immunization programmes and place even more lives at risk. Public acceptance of vaccination is highly dependent upon the quality of vaccines used. A well-functioning NRA skilled in necessary regulatory processes is essential if graduating countries choose to self-procure their vaccines.

The transition planning assessments found significant shortfalls in the strength of NRA, and recommended improvement measures to take place over the graduation period. In Georgia, e.g. the team recommended that the NRA be strengthened to enable it to register and monitor the competitive behaviour of local agents representing vaccine manufacturers, and to track and respond rapidly and effectively to reports of any adverse events following immunization.

Graduating countries have benefited from inter-country exchanges, knowledge sharing workshops and regular visits by senior GAVI, WHO, UNICEF and other officials, raising the political profile of immunization. These global organizations have also supported countries with technical assistance, including for immunization planning, surveillance, communication, Effective Vaccine Management programmes and NRA development. Once GAVI support ends, the gains made from such visibility and outside financial support and technical assistance could suffer, unless local advocacy efforts are intensified and national technical skills are strengthened. At present, most immunization-related technical support from global partner institutions is focused on GAVI countries, with little support for graduated and other middle-income countries.

Latin America is an important exception. The Pan American Health Organization (PAHO) backs immunization advocacy efforts and provides technical assistance for large numbers of middle-income countries in the Western Hemisphere. It organizes ‘Vaccination Week in the Americas’ with high profile events in PAHO countries. Its ProVac initiative helps to strengthen countries’ technical capacity to make evidence-based decisions on new vaccine introductions (PAHO 2013). Similar efforts could be mounted in other regions.

Graduating countries will need to consider whether and when to introduce other new vaccines entering the market, such as for malaria and cholera. Some countries such as Mongolia have established an independent National Immunization Technical Advisory Group (NITAG) to advise the government on evidence-based immunization policy and programme decisions. Other graduating countries visited have not yet moved in this direction.

To assist countries in making these decisions, graduating countries will need better access to data on vaccine efficacy and immunization costs, price and other market information, and relevant analytical tools (Kaddar *et al.* 2013). Several ongoing projects could help, including WHO’s Vaccine Product Price and Procurement (V3P) project,^5^ the Supporting Independent Immunization and Vaccine Advisory Committees (SIVAC) Initiative’s support to develop NITAGs in various countries, and PAHO’s ProVac project.

## Conclusion

Graduation is a test of GAVI’s business model, including its eligibility and co-financing policies and market shaping activities. The GAVI Alliance and its partners are working to position graduating countries for success in assuming full financial responsibility and management of vaccine procurement and regulation, and in developing the internal capacity for vaccine policy analysis and advocacy.

GAVI and the UNICEF SD are also negotiating on behalf of graduating countries with manufacturers to obtain access to low prices, which would help countries with predictable financial requirements and greater affordability of new vaccines. As mentioned earlier, GAVI prices have been secured for some graduating countries from suppliers of pneumococcal, pentavalent and rotavirus vaccines. GAVI is now considering ways in which it can standardize these arrangements, ideally obtaining GAVI prices for graduating countries for a number of years after they graduate, as well as affordable prices for other lower-middle-income countries.

Most current graduating countries are expected to experience strong and sustained economic growth, which should provide relatively favourable conditions for financial self-sufficiency in vaccines and immunization services—as long as there is adequate political commitment and technical capacity to plan and manage implementation of their immunization programmes.

It is also important to consider both the absolute size of additional funding needed and the demand this will place on the health budget, plus the fiscal challenges of year-on-year increases, which could be large in cases where current domestic funding for immunization is small. Such large increases in government spending may be difficult for countries to achieve. However, other countries with strong commitment to immunization have documented success in increasing public spending for new vaccines in a short time period. In South Africa, for example, the government introduced pneumococcal and rotavirus vaccines in 2008. The high cost of these new vaccines caused the share of the health budget devoted to immunization to triple from 0.3% to 0.9% in a short period (Blecher *et al.* 2012).

The magnitude of increase in domestic funding will depend on the number of new vaccines a country is introducing. Although not observed in the five focus countries, some countries may be tempted to introduce as many new vaccines as possible in the year prior to graduation, in order to capitalize on GAVI funding that will decline soon thereafter. It may therefore be prudent for GAVI to consider providing more time for a country to apply and conduct affordability analyses *ex ante* before approving applications for new vaccines from countries which are close to graduation.

Transition planning needs to look beyond the limited number of GAVI-financed new vaccines and take into account the funding requirements for the entire national portfolio of vaccines, as well as non-vaccine immunization costs (for personnel, transport, cold chain, surveillance, quality assurance, etc.). The importance of projecting the full financial burden of graduation for countries highlights the need for more accurate non-vaccine delivery cost tracking and estimates, and the inclusion of these costs in future analyses.

In addition to financial sustainability, the graduation planning exercises identified a number of other challenges that could impact the ability of countries to successfully graduate from GAVI support. These include vaccine procurement policies and practices, market intelligence (forecasted prices, expected entry of new suppliers and vaccine products, etc.), national regulatory capacity, and immunization technical advisory bodies and their effective functioning.

Lessons learned from the graduation process and from transition planning will be helpful for future graduating countries. Timor Leste crossed the eligibility threshold in 2013 and Nicaragua, Papua New Guinea and Uzbekistan are to start the graduation process in January 2014. Future countries will have lower per capita incomes and larger birth cohorts than some of the current graduates, and may have a larger number of vaccines to sustain, making domestic financing challenging.

The country assessments conducted in 2012 have deepened the understanding by GAVI, technical partners, and graduating countries of the issues and possible solutions for successful graduation. GAVI is currently the main funder of technical support for country-level immunization programmes (channelled through WHO and other agencies). GAVI may need to change its business model or have other partners come forward to help build the enabling environment and provide targeted technical support to graduated and other middle-income countries’ immunization programmes. The PAHO region provides some important examples (PAHO 2013), illustrated in the previous section of this article, of what can be done in this regard. In addition, it is important that graduating countries are able to learn from each other’s experiences. GAVI could play a role in convening graduating countries for such joint learning.

GAVI’s experience in assessing the readiness and needs of the current wave of graduating countries is helping to inform its future policies on country eligibility and graduation. It is expected that over the next few years, GAVI will consider a range of additional instruments—financial and non-financial—that could ensure a smooth and sustainable graduation process.

Other global health initiatives in areas such as AIDS, malaria and family planning, which have also relied on external funding, are exploring ways to help countries move to domestic financial self-sufficiency and achieve full operational independence. They may be able to learn from GAVI’s experience with graduation and transition planning. At the same time, GAVI and its graduating countries may benefit from exchanges of experience with organizations such as the Global Fund and PEPFAR, which are striving to reduce the dependency of middle-income countries on their financing and to promote a smooth transition to increased national funding and ‘ownership’ (Global Fund to Fight AIDS, Tuberculosis and Malaria 2011; PEPFAR 2013).

## Endnotes

^1^The World Bank’s highly concessional International Development Association (IDA) credits, however, have a lower eligibility threshold than GAVI’s threshold. This was $1195 GNI per capita in 2013. The Bank uses lack of creditworthiness along with the income threshold to qualify countries for IDA credits (http://www.worldbank.org/ida/what-is-ida/fund-for-the-poorest.pdf, accessed 15 October 2013).

^2^Comprehensive multi-year plans for immunization, poverty reduction strategy plans, medium term expenditure frameworks and health sector strategies.

^3^Including UNICEF Supply Division, Results for Development Institute and the Sabin Institute Sustainable Immunization Financing Project.

^4^Although countries receive GAVI-supported vaccines, they obtain ‘GAVI prices’ through the UNICEF SD. Because of significant volumes of vaccines procured on behalf of low- and lower-middle-income countries and guaranteed funding by donors, manufacturers have provided vaccines at a low-pricing tier, sometimes at a fraction of the price in high-income countries.

^5^This WHO project aims to identify and establish appropriate, comprehensive mechanisms to provide countries with accurate, useful data on vaccine products, prices and procurement methods (World Health Organization 2013).
